# Prevalence of higher-grade dysplasia in persistently high-risk human papillomavirus positive, cytology negative women after introduction of the new cervical cancer screening in Germany

**DOI:** 10.1007/s10552-023-01677-z

**Published:** 2023-02-28

**Authors:** Laura Berger, Maja Wolf-Breitinger, Christel Weiß, Benjamin Tuschy, Sebastian Berlit, Marc Sütterlin, Saskia Spaich

**Affiliations:** 1 Department of Obstetrics and Gynecology, University Medical Center Mannheim, Heidelberg University, Theodor-Kutzer Ufer 1-3, 68165 Mannheim, Germany; 2 Department of Medical Statistics and Biomathematics, Medical Faculty Mannheim, Heidelberg University, Mannheim, Germany

**Keywords:** hrHPV, CIN, VaIN, Colposcopy, Negative cytology, Cervical cancer screening

## Abstract

**Purpose:**

According to the recently implemented organized cervical cancer screening program in Germany, women older than 35 years with negative cytology but persistent high-risk human papilloma virus (hrHPV) infection > 12 months should be referred to colposcopy for further evaluation. This study aimed to present and dissect colposcopic and histopathological findings with particular focus on associated hrHPV genotypes.

**Methods:**

This study is a retrospective analysis of clinical data from 89 hrHPV positive patients with normal cytology who underwent colposcopic examination at a certified dysplasia outpatient clinic in Germany in 2021.

**Results:**

While 38 (43%) women had a normal colposcopic finding, 45 (51%) had minor and 6 (7%) major changes. Thirty-one (35%) of the women were HPV 16 and/or HPV 18 positive and 58 (65%) women were positive for other hrHPV only. Among patients who underwent colposcopy with biopsies (in case of an abnormal finding or type 3 transformation zone, *n* = 68), eight (12%) had cervical intraepithelial neoplasia (CIN) 3 and six (9%) had CIN 2. The proportion of women diagnosed with CIN 3 varied among different hrHPV genotypes (HPV 16: 11%, HPV 18: 33%, HPV 31: 27%, HPV 33: 33%, HPV 52: 33%).

**Conclusion:**

Persistently hrHPV positive women with negative cytology are at increased risk of being diagnosed with CIN 3. As CIN 3 prevalence seems to differ with regard to hrHPV strain, immediate HPV genotyping for risk stratification and subsequent early referral for colposcopy might constitute a feasible strategy.

## Introduction

The recognition that a persistent HPV infection is causative for the vast majority of cervical cancers has led to a transition from cytology-based to HPV-based screening programs in an increasing number of countries, either as a stand-alone screening method or in combination with cytology (co-testing) [1, 2]. Several large randomized controlled trials comparing HPV-based screening with cytology have shown that HPV-based screening is more effective in detecting CIN 3 in the first screening round with a reduced occurrence of CIN 3 in the second round of screening, thus resulting in earlier detection of clinically relevant precancerous lesions [3–7]. Additionally, a pooled analysis of follow-up data of four European randomized controlled trials demonstrated that HPV-based screening provides 60–70% greater protection against invasive cervical cancer compared with cytology alone [8]. However, HPV testing lacks specifity for high-grade CIN especially in young women, as many HPV infections are transient in nature and clear on their own within 24 months after exposure [9]. Due to the higher sensitivity, HPV testing is associated with a higher referral rate for colposcopy [10, 11] and subsequent risk for overtreatment of regressive CIN 2, that may result in adverse obstetric outcomes and other treatment-related complications such as pain, bleeding or postoperative cervical stenosis [5, 12–15]. Strategies for limiting the higher rate of colposcopic referral could be performing cytological triage of hrHPV women or instant HPV genotyping [11, 16–19].

In January 2020, almost 50 years after the introduction of an annual opportunistic cytology screening for cervical cancer in Germany, a nationwide organized screening program was implemented. According to the age-dependent screening algorithm, women between 20 and 34 years of age are entitled to an annual cytological examination, whereas women ≥ 35 years should be screened by co-testing with HPV testing and cytology every 3 years. Women with negative cytology (Pap I) and a repeatedly positive HPV test in re-screening after 12 months should be referred to colposcopy within 3 months [20].

There is increasing evidence that cervical cancer risk is strongly associated with the underlying hrHPV genotype, revealing that HPV 16 poses the highest risk, followed by HPV 18, HPV 31 and HPV 33 [18, 21–26]. As revealed by a large meta-analysis, overall and type-specific hrHPV prevalence considerably differs across geographical regions with Africa having the highest and Southeastern Asia the lowest overall hrHPV prevalence. While in most regions of the world HPV 16 had the highest type-specific prevalence, there were considerable differences regarding the second most frequent type in cytology negative women (Northern and Western Europe HPV 18, Southern Europe HPV 66, Eastern Europe HPV 31, South America HPV 58, Northern America HPV 53) [27]. Among cytology negative, hrHPV positive women in Germany, to our knowledge there are no data regarding the genotype specific hrHPV prevalence and simultaneous analysis of colposcopy-guided biopsy results. As a consequence of the recently implemented cervical cancer screening program in Germany, identification of cytology negative, hrHPV positive women will increase steadily and thus the need for reasonable and feasible management strategies to enhance program effectiveness and limit burden on healthcare resources. Therefore, the objective of this study was to present colposcopic and histopathological findings among this population of women with particular focus on the underlying hrHPV genotype.

## Materials and methods

This study is a retrospective analysis of hrHPV positive women with negative cytology (Pap I) who attended the certified dysplasia outpatient clinic at University Medical Center Mannheim, Heidelberg University, Germany between January and December 2021. All included women were referred for colposcopy due to a persistently positive HPV test (repeated co-testing after 12 months) after attending the newly introduced cervical cancer screening program according to the predefined diagnostic algorithm. Exclusion criteria were defined as pregnancy, history of total hysterectomy and history of conization with positive resection margins. The patient data including colposcopic and histopathological findings, result of HPV testing and HPV vaccination status were obtained from the patient chart. Data on hrHPV genotype were available in different levels of exactness depending on the detection method applied: exact identification of hrHPV genotype using a multiplex PCR assay (f-HPV typing™ Kit, Genomed Ltd, Middlesex, United Kingdom) or partial genotyping using the cobas*®* HPV test [exact identification of HPV 16 and HPV 18 and pooled analysis of 12 other hrHPV types (31, 33, 35, 39, 45, 51, 52, 56, 58, 59, 66 and 68)] [28]. The presence of a co-infection was analyzed in patients for whom exact identification of hrHPV genotype was available (*n* = 63) and for patients who were tested using cobas*®* HPV test (Roche Diagnostics, Mannheim, Germany) with a positive result for both HPV 16/18 and other hrHPV (*n* = 1).

Colposcopic findings of the cervix and vagina were classified according to the 2011 colposcopic terminology of the International Federation for Cervical Pathology and Colposcopy (IFCPC) [29]. Patients who had an abnormal colposcopic finding (minor or major change) underwent punch biopsy of the macroscopically visible lesion(s). If the colposcopic examination was considered normal and the transformation zone was visible, no biopsies were obtained. In case the squamocolumnar junction was not visible (transformation zone (TZ) type 3), an endocervical curettage (ECC) was performed, if technically possible.

This study was approved by the Ethics Committee II of the University of Heidelberg, Medical Faculty Mannheim (2022-808-AF 11).

## Statistics

All data were recorded in a Microsoft Excel spreadsheet. Statistical analysis was performed using SAS® software (release 9.4, SAS Institute Inc., Cary, NC, USA, www. sas.com). Categorial data are presented as absolute and relative frequencies. For quantitative normally distributed data arithmetic mean and standard deviation are given. Fisher’s exact test was used to compare the prevalences of cervical dysplasia between HPV 16/18 positive and other hrHPV positive patients. A *p*-value < 0.05 was considered statistically significant. Positive predictive values (PPVs) for the most frequently observed HPV strains were calculated to analyze diagnostic performance of HPV testing for detection of high-grade dysplasia.

## Results

The data of 89 women were included in this analysis. The mean age of the study population was 49 ± 11 years. Regarding HPV vaccination status, only one patient (1%) was fully vaccinated and one patient (1%) was partially vaccinated. Since the vast majority of women (98%) was not vaccinated, the vaccination status was not taken into account for further analyses.

The distribution of different hrHPV genotypes among the study population (*n* = 89) and the respective infection pattern (single infection or co-infection) is presented in Table 1. The most frequent genotype was HPV 16 (26%, *n* = 23), followed by HPV 31 in 15% (*n* = 13) of the cases. Each of the genotypes HPV 18 and HPV 52 was observed in 9% (*n* = 8) of the patients. 26 patients (29%) were tested positive for pooled hrHPV (other). Since women with multiple infections are counted separately for each HPV type, the sum of the individual HPV types surmounts the total number of included women. On a superordinate level, 31 (35%) of the women were HPV 16/18 positive (HPV 16 + and/or HPV 18 +) and 58 (65%) women were tested positive for other hrHPV only (HPV 16-/HPV 18-/other hrHPV +). Women positive for HPV 16/18 tended to be older than women positive for other hrHPV only, however, this was not statistically significant (HPV 16/18: 52 ± 11 years, other hrHPV: 47 ± 11 years, *p* = 0.0967). Considering the exactly genotyped hrHPV cases, a single infection was present in 84% (54/64) of the women. In 16% (10/64) of the women, a co-infection with different hrHPV strains was detected (in 9 women with two different strains, in one woman with four different strains).

**Table 1:** Distribution of different hrHPV genotypes (single and co-infection) in hrHPV positive, cytology negative women (*n* = 89)

HPV type	Total	Single infection	Co-infection
16	23 (26%)	19 (21%)	4 (5%)
18	8 (9%)	6 (7%)	2 (2%)
31	13 (15%)	9 (10%)	4 (5%)
33	5 (6%)	2 (2%)	3 (3%)
35	1 (1%)	0	1 (1%)
39	4 (5%)	1 (1%)	0
45	4 (5%)	3 (3%)	1 (1%)
51	4 (5%)	3 (3%)	1 (1%)
52	8 (9%)	5 (6%)	3 (3%)
56	1 (1%)	0	1 (1%)
59	3 (3%)	2 (2%)	1 (1%)
66	1 (1%)	1 (1%)	0
Other hrHPV not further specified	26 (29%)		

Table 2 shows the colposcopic findings and corresponding biopsy results. In total, 38 women (43%) had a normal colposcopic result, 45 (51%) had minor and six (7%) had major changes. In 19 of the women (21%), a cervical biopsy was not performed—either it was omitted due to a normal colposcopic finding and transition zone 1 or 2 (*n* = 11, 12%) or it was unsuccessful due to atrophic changes resulting in cervical stenosis (*n* = 8, 9%). In two women (2%), the obtained biopsy was categorized as non-representative by the pathologist.

**Table 2:** Colposcopic and histopathological findings in hrHPV positive, cytology negative women (*n* = 89)

Colposcopic finding	Histopathological result	Number of women (*n* = 89)
Normal		**38 (43%)**
•TZ type 1 or 2	Not obtained	11 (12%)
•TZ type 3		27 (30%)
	No dysplasia	16 (18%)
	CIN 1	0 (0%)
	CIN 2	0 (0%)
	CIN 3	1 (1%)
	VaIN 3	1 (1%)
	Not representative	1 (1%)
	ECC not possible due to stenosis/atrophy	8 (9%)
Minor		**45 (51%)**
	No dysplasia	30 (34%)
	CIN 1	3 (3%)
	CIN 2	5 (6%)
	CIN 3	5 (6%)
	VaIN 3	1 (1%)
	Not representative	1 (1%)
Major		**6 (7%)**
	No dysplasia	3 (3%)
	CIN 1	0 (0%)
	CIN 2	1 (1%)
	CIN 3	2 (2%)
	VaIN 3	0 (0%)
	Not representative	0 (0%)

Out of all women who underwent representative biopsy (*n* = 68), three women (4%) had CIN 1, six women (9%) had CIN 2, eight women (12%) CIN 3 and two women (3%) vaginal intraepithelial neoplasia (VaIN) 3. No case of invasive cancer was detected. Higher prevalences for CIN 2 + and CIN 3/VaIN 3 were found in the HPV 16/18 group compared to the other hrHPV group, albeit this difference was not statistically significant (prevalence of CIN 2 +: HPV 16/18 group 36% vs. other hrHPV group 16%, *p* = 0.0805. Prevalence of CIN 3/VaIN 3: HPV 16/18 group 24% vs. other hrHPV group 9%, *p* = 0.1543). (see Table 3). The age of patients with and without higher-grade cervical dysplasia (CIN 2 +) as well as the age of patients with and without high-grade dysplasia (CIN 3/VaIN 3) did not differ significantly (normal histological finding and CIN 1: 47 ± 12 years vs. CIN 2 +: 47 ± 11 years, *p* = 0.7921. Normal histological finding and CIN 1/2: 48 ± 11 years vs. CIN 3/VaIN 3): 46 ± 11 years, *p* = 0.5756).

**Table 3:** Prevalence of cervical dysplasia stratified by HPV 16/18 + and other hrHPV + cases in cytology negative patients with abnormal colposcopy or normal colposcopy and TZ type 3 (*n* = 68, two patients with unrepresentative biopsy results were excluded)

	Total	HPV 16/18^a^	Other hrHPV^b^	*p*-value
Normal	49 (72%)	16 (64%)	33 (77%)	0.2769
CIN 1 +^c^	19 (28%)	9 (36%)	10 (23%)	0.2769
CIN 2 +^d^	16 (24%)	9 (36%)	7 (16%)	0.0805
CIN 3/VaIN 3	10 (15%)	6 (24%)	4 (9%)	0.1543
	*n* = 68	*n* = 25	*n* = 43	

^a^Includes cases positive for HPV 16 and/or HPV 18

^b^Indicates HPV 16-/HPV18-/other hrHPV positive cases

^c^Cervical intraepithelial neoplasia grade 1 or worse (including VaIN 3)

^d^Cervical intraepithelial neoplasia grade 2 or worse (including VaIN 3)

Table 4 displays the histopathological results of the women who underwent representative biopsy (*n* = 68) stratified by hrHPV genotype. Out of the 19 patients who were positive for HPV 16, three (16%) had CIN 2, two (11%) had CIN 3 and two (11%) had VaIN 3. Two out of the six patients (33%) positive for HPV 18 had CIN 3. Six cases of CIN 3 (9%) occurred in patients with a single HPV infection, while two cases of CIN 3 (3%) were recorded in patients with multiple infections (types 33, 52 and types 18, 31, 52, 56). In one patient with CIN 3 no exact identification of HPV genotype was available. In our study population, women positive for HPV 35, HPV 51, HPV 59 or HPV 66 did not show dysplastic changes.

**Table 4:** Histopathological results by different hrHPV genotypes in cytology negative patients with abnormal colposcopy or normal colposcopy and TZ type 3 (*n* = 68, two patients with unrepresentative biopsy results were excluded)

HPV type	Total	CIN 1	CIN 2	CIN 3	VaIN 3
16	19/68 (28%)	–	3/19 (16%)	2/19 (11%)	2/19 (11%)
18	6/68 (9%)	–	–	2/6 (33%)	–
Other hrHPV not further specified	15/68 (22%)	2/15 (13%)	1/15 (7%)	1/15 (7%)	–
31	11/68 (16%)	–	2/11 (18%)	3/11 (27%)	–
33	3/68 (4%)	–	–	1/3 (33%)	–
35	1/68 (1%)	–	–	–	–
39	4/68 (6%)	–	1/4 (25%)	–	–
45	4/68 (6%)	1/4 (25%)	–	–	–
51	4/68 (6%)	–	–	–	–
52	6/68 (9%)	–	–	2/6 (33%)	–
56	1/68 (1%)	–	–	1/1 (100%)	–
59	2/68 (3%)	–	–	–	–
66	1/68 (1%)	–	–	–	–

Positive predictive values of HPV testing for the presence of high-grade vaginal and cervical dysplasia dependent on the HPV strain ranged from 16% (95% CI 7–31) to 46% (95% CI 17–77) for CIN 2 + and 9% (95% CI 3–22) to 33% (95% CI 4–78) for CIN 3/VaIN 3. Highest PPVs for CIN 2 + were observed for HPV 31 (46%, 95% CI 17–77) and HPV 16 (37%, 95% CI 16–62) and for CIN 3/VaIN 3 for HPV 18 and HPV 52 (both 33%, 95% CI 4–78). Lowest PPVs were seen for other hrHPV both for CIN 2 + (16%, 95% CI 7–31) and CIN 3/VaIN 3 (9%, 95% CI 3–22) (see Table 5).

**Table 5:** Positive predictive values of HPV testing for detection of high-grade dysplasia in cytology negative patients with abnormal colposcopy or normal colposcopy and endocervical curettage performed due to TZ type 3

	CIN 2 + PPV (95% CI)	CIN 3/VaIN 3 PPV (95% CI)
HPV 16^a^	37 (16–62)	21 (6–46)
HPV 18	33 (4 –78)	33 (4–78)
HPV 16/18^b^	36.(18–58)	24 (9–45)
Other hrHPV^c^	16 (7–31)	9 (3–22)
HPV 31	46 (17–77)	27 (6–61)
HPV 52	33 (4–78)	33 (4–78)

^a^Cases positive for HVP 16 alone (single infection) or in combination with another HPV strain (co-infection) (analogous definition of HPV 18, HPV 31 and HPV 52)

^b^Cases positive for HPV 16 and/or HPV 18

^c^HPV 16-/HPV18-/other hrHPV positive cases

## Discussion

This retrospective single-center study provides colposcopic and histopathological results from cytology negative, repeatedly hrHPV positive women. Due to the recently implemented organized cervical cancer screening in Germany, this population of women will continue to rise in number and hence requires increasing attention in developing effective and pertinent management strategies.

### Prevalence of hrHPV genotypes

Considering the distribution of different hrHPV strains, we found HPV 16 to be prevalent most frequently (26%), followed by HPV 31 (15%) and both HPV 52 and HPV 18 (9%). HPV 39, HPV 45 and HPV 51 all had a prevalence of 6%, the other hrHPV types were below 5%. To a larger extent, this distribution is similar to data from a Danish cohort study reporting HPV 16 as the most common type with a comparable frequency to ours (24.2%), also followed by HPV 31 (20.5%) and HPV 52 (20.1%) and with lesser frequency by HPV 51 (14.4%), HPV 33 (13.4%), HPV 39 (11.3%), HPV 45 (11.0%), HPV 56 (10.0%) and HPV 18 (9.5%) [21]. A similar order was also observed in another more recent cohort study from Denmark [30]. It should be kept in mind that in our study no exact HPV genotype was available for 29% of the patients (other hrHPV positive), suggesting that true proportions of non HPV 16/18 positives will be correspondingly higher.

### Colposcopic results

In our study, 43% of the patients had a normal colposcopic result, 51% had minor and 7% had major changes. Data on colposcopic findings among cytology negative, hrHPV positive women are scarce, as the vast majority of studies focus on reporting of colposcopy-guided biopsy results, but not on the colposcopic result itself. In a study by del Pino et al. evaluating the accuracy of initial colposcopy to predict progression to higher-grade CIN, among the cytology negative, hrHPV positive subgroup, 60% of the women had a normal colposcopy, 37% had minor and 3% major changes. However, comparability of those results to ours is limited, as in the aforementioned study all patients who had a diagnosis of CIN 2 or CIN 3 in the biopsy at enrolment were excluded. Interestingly, del Pino et al. found that hrHPV positive women with normal, borderline, or low-grade abnormalities in cytology had a similar risk of progression to high-grade lesions and that the initial colposcopy findings did not provide relevant information on the risk of progression [31]. Generally, diagnostic accuracy of colposcopy is still controversial with sensitivity for CIN 2 + ranging from 30 to 60% [32–34]. To improve diagnostic accuracy, several studies suggest that increasing the number of biopsies and obtainment of random biopsies from normal appearing areas seems to be beneficial [35–40]. In our study, we did not take random biopsies from macroscopically disease-free areas and refrained from performing an ECC when the squamocolumnar junction was visible, which is consistent with the approach proposed by the American Society of Colposcopy and Cervical Pathology (ASCCP) and the German evidence- and consensus-based (S3) guideline on the prevention of cervical cancer [41, 42]. A study on other hrHPV positive, cytology negative women by Kabaca et al. showed that ECC specimens contained CIN 1 in 4.3% of the patients, CIN 2 in 1.2% and CIN 3 in 0.8%. According to their study protocol, ECC specimens and cervical biopsy were mandatorily obtained from all included patients. However, the authors did not provide information on the proportion of the abnormalities which were found by ECC alone and of those where ECC and cervical biopsy yielded the same result (representing cases in which ECC did not provide an extra benefit) [43].

### Histopathological results

While we observed only very few CIN 1 cases (three in total, all of them other hrHPV positive), prevalence of CIN 1 is reported as high as 21.6% in HPV 16/18 positive women [44] and 23.9% for other hrHPV positive, cytology negative women [43]. The comparably low number of CIN 1 cases in our study might at least be partially explained by referral for colposcopy only in case of a persistently positive HPV test after 12 months. According to a large meta-analysis, regression rate of CIN 1 was around 60%, persistence rate was around 25% and progression rate to CIN 2 and worse or CIN 3 were 11 and 2%, respectively [45].

In our study population, 12% of the women with discordant co-testing from whom a biopsy was obtained had CIN 3 (equaling 9% of the entire study population including patients with normal colposcopy). This is well in line with the recently published retrospective findings by Peace et al. reporting of 12.0% CIN 3 in a cytology negative, HPV positive collective with abnormal colposcopic results. However, compared to our study population Peace et al. followed the 2012 ASCCP screening algorithm recommending an immediate referral to colposcopy for HPV 16 or HPV 18 positive cases and repetition of co-testing only for other HPV positive cases [46]. Prospective data from the German WOLPHSCREEN study by Luyten et al. revealed a CIN 3 + prevalence of 12.9% among the cytology negative, hrHPV positive subgroup. As in our study, colposcopy was performed if women remained hrHPV positive after repeated testing at 12 months. Since in WOLPHSCREEN in case of a normal colposcopic result no biopsies were taken, their CIN 3 + prevalence is slightly higher than the 9% CIN 3 observed within our corresponding population (i.e., in relation to our entire study population including all patients with normal colposcopy of whom no biopsy was obtained) [47].

In a large Danish prospective cohort study with a follow-up time of up to 11.5 years, 9.7% of the hrHPV positive women with negative cytology at baseline developed CIN 3 + during the observational period. Stratified by hrHPV type and type of infection (single or multiple infection), the highest proportion of women who developed CIN 3 + was observed among those with a single HPV 16 infection (23.3%), followed by those with a single HPV 33 infection (17.9%) or single HPV 31 infection (11.3%). Interestingly, when HPV 16 occurred as a co-infection with another hrHPV type, the proportion of women developing CIN 3 was only 17.2% and for HPV 31 positives in conjunction with another hrHPV type except HPV 16 only 9.7%. Regarding HPV 18, the proportion of women developing CIN 3 did not differ between solely or co-infected women (single: 10.8%, co-infection 11.4%) [22]. In the literature, the effect of co-infections in cervical carcinogenesis remains controversial. A large Chinese cross-sectional study corroborates the aforementioned observation showing that women with a single HPV 16 infection are at higher risk for the progression to CIN 3 + compared to a concurrent co-infection with other hrHPVs, proposing a yet unclear antagonistic interaction [48]. On the other hand, there is evidence that multiple infections may act synergistically in cervical carcinogenesis [49, 50]. In our study, the majority of CIN 3 cases was observed in patients with single infections. Yet due to the limited number of included patients, further analysis of the contribution of the type of infection is beyond the scope of this trial.

In our study, prevalence of CIN 3 was 14% for HPV 16/18 positive women who underwent biopsy and 9% for other hrHPV positive. Reviewing literature, CIN 3 prevalence for the former subgroup ranges from 9.1 to 11.8% [44, 46, 51] and from 2.7 to 14.6% for the latter [43, 46, 51]. However, comparability of the studies among each other and with ours is very limited due to various reasons such as differences in study design (retrospective or prospective), timing of colposcopy (immediate or only in case of repeatedly positive HPV testing at 12 months), threshold for obtaining histological specimens (omission of biopsy in case of normal colposcopic finding or taking a biopsy and/or ECC regardless of colposcopic finding), considered set of patients (all hrHPV positive patients or only those from whom a biopsy was obtained), median age of the study population, method applied for HPV testing and regional prevalence of particular hrHPV types.

In our study population, among the HPV 16 positive women, 11% had CIN 3 and among the HPV 18 positives, 33% had CIN 3. Peace et al. reported a CIN 3 + prevalence of 14.7% among HPV 16 positive cases, yet only of 1.4% among HPV 18 positives [46]. Including only other hrHPV positive cases, Kabaca et al. observed a CIN 3 + prevalence of 8.3% for HPV 33 positives, 7.8% for HPV 31 positives and 5.1% for HPV 39 positives [43]. Compared to Kabaca et al., the CIN 3 prevalence for distinct hrHPV strains in our study collective was a lot higher (HPV 33: 33%, HPV 31: 27%, HPV 52: 33%). This might be at least partially explained by the fact that in our study cases with multiple infections were counted for each HPV strain separately, while Kabaca et al. did not provide insight into their approach of handling the occurrence of multiple infections. However, in our study the prevalence of higher-grade dysplasia stratified according to HPV subtype needs to be interpreted with caution due to the small number of patients and single-center character. Moreover, direct comparison of PPVs calculated for individual hrHPV genotypes within our population with data by Giray et al. showed that PPVs calculated for our population were generally higher. We observed PPVs for the detection of CIN 2 + up to 46% for HPV 31, while the corresponding PPV by Giray et al. was 20%. CIN 2 + PPV in our population for HPV 16 was 37% and for HPV 18 33% as compared to 16.4 and 11.7% in the study by Giray et al. The relatively high PPV of HPV 16 in our study, even though we only observed two cases of CIN 3 among HPV 16 positives, is explained by three cases of CIN 2 and two cases of VaIN 3 within this subgroup. It should be borne in mind that PPVs depend on the prevalence of the target condition in the study population, which complicates the comparison of PPVs between different studies.

We decided to include the cases of VaIN 3 into our study population, as it also is a HPV-associated condition in most cases and the direct precursor of vaginal carcinoma. VaIN is difficult to diagnose as it is a very rare and mostly asymptomatic condition. It can occur alone or simultaneously with cervical or vulvar intraepithelial neoplasia. It has been reported that there is a positive correlation between the grade of VaIN and grade of concomitant CIN, although there is a non-negligible number of women in whom vaginal lesions were found to be more severe than cervical lesions [52]. As especially HPV 16 and HPV 31 were found to be highly associated with high-grade VaIN, it is advisable to be particularly attentive for signs of VaIN among patients tested positive for these hrHPV subtypes [53]. However, to add another degree of complexity, recent data suggests that concomitant CIN and VaIN in the same patient develop independently, since in the great majority of cases different hrHPV types were identified within the cervical and vaginal lesions [54]. Both patients who were diagnosed with VaIN 3 in our study were HPV 16 positive and did not have any cervical abnormalities.

Even though we did not detect any cases of invasive cervical carcinoma, other groups found invasive cervical carcinoma in up to 0.5% of the women [43, 44, 46]. Interestingly, Giray et al. observed that the rate of invasive cancer did not differ significantly between women with normal and abnormal cytology in HPV 16/18 positive women (0.5% vs. 1.5%, *p* = 0.082) and concluded that Pap testing could be unnecessary in HPV 16/18 positive women who will undergo colposcopy-guided biopsy to diagnose invasive cervical cancer [44].

For countries undergoing substantial changes in cervical cancer screening, such as Germany, ongoing evaluation of screening results and program effectiveness is essential to highlight any areas for improvement. As hrHPV positive women with negative cytology who have a considerable risk of being diagnosed with CIN 3 are now identified at constantly increasing numbers, refinement of management strategies for this subgroup is crucial. In line with the 2019 ASCCP consensus guidelines, future approaches might comprise a personalized risk-based management, since the individual risk for having or developing CIN 3 is dependent on a combination of past history and current results. For example, a new abnormal test results within 5 years after a negative HPV test or co-test reduces the estimated risk for CIN 3 + by approximately 50%. Thus, incorporating the patient’s history of previous HPV test results into clinical decision-making might help to distinguish high-risk from low-risk patients and subsequently adapt management recommendations [41]. Besides characterization of the infection (duration of infection and responsible HPV type), further risk stratification methods might include the implementation of biomarkers, such as p16/ki67 dual staining or viral or host cell DNA methylation assays as well as the consideration of HPV vaccination status [55].

## Limitations

There are several limitations to our study that should be noted. Due to the retrospective design it is possible that cases might have been missed, so there is no claim for completeness of the cohort. Furthermore, data regarding HPV genotyping was incomplete. Another shortcoming is the limited number of patients included, which might reduce the statistical power of the results. On the one hand, this is due to the study period of 12 months, which was chosen to enable the provision of current data regarding the recently introduced new cervical cancer screening program in Germany. On the other hand, the limited number of included patients is owed to the single-center design. Therefore, a multi-center prospective study with adequate follow-up is required to further explore the risk of developing high-grade CIN among cytology negative, hrHPV positive patients.

## Conclusion

Our results suggest that women with discordant co-testing have a significant risk of being diagnosed with CIN 3. As the risk for developing a high-grade CIN might be associated with the underlying hrHPV type, employing instant HPV genotyping and, if necessary, immediate referral for colposcopy may result in substantial improvement in screening management.

## Data Availability

The datasets generated during and/or analyzed during the current study are available from the corresponding author on reasonable request.
